# Physiological and transcriptome analysis reveals that prohexadione-calcium promotes rice seedling’s development under salt stress by regulating antioxidant processes and photosynthesis

**DOI:** 10.1371/journal.pone.0286505

**Published:** 2023-06-14

**Authors:** Yao Li, Hang Zhou, Naijie Feng, Dianfeng Zheng, Guohui Ma, Shengjie Feng, Meiling Liu, Minglong Yu, Xixin Huang, Anqi Huang

**Affiliations:** 1 College of Coastal Agriculture Sciences, Guangdong Ocean University, Zhanjiang, Guangdong, China; 2 Agricultural College, Heilongjiang Bayi Agricultural University, Daqing, Heilongjiang, China; 3 Shenzhen Research Institute, Guangdong Ocean University, Shenzhen, Guangdong, China; 4 South China Center, National Salt-alkali Tolerant Rice Technology Innovation Center, Zhanjiang, Guangdong, China; ICAR - National Research Center on Plant Biotechnology, INDIA

## Abstract

Prohexadione-calcium (Pro-Ca) has been proved to play an important role in releasing abiotic stress in plants. However, there is still a lack of research on the mechanism of Pro-Ca alleviating salt stress in rice. To explore the protective effects of Pro-Ca on rice seedlings under salt stress, we investigated the effect of exogenous Pro-Ca on rice seedling under salt stress by conducting the following three treatment experiments: CK (control), S (50 mmol·L^−1^ NaCl saline solution) and S + Pro-Ca (50 mmol·L^−1^ NaCl saline solution + 100 mg·L^−1^ Pro-Ca). The results indicated that Pro-Ca modulated the expression of antioxidant enzyme-related genes (such as *SOD2*, *PXMP2*, *MPV17*, *E1*.*11*.*1*.*7*). Spraying Pro-Ca under salt stress significantly increased in ascorbate peroxidase, superoxide dismutase, and peroxidase activity by 84.2%, 75.2%, and 3.5% as compared to the salt treatment, as demonstrated by an example of a 24-hour treatment. Malondialdehyde level in Pro-Ca was also dramatically decreased by 5.8%. Moreover, spraying Pro-Ca under salt stress regulated the expression of photosynthesis genes (such as *PsbS*, *PsbD*) and chlorophyll metabolism genes (*heml*, *PPD*). Compared to salt stress treatment, spraying Pro-Ca under salt stress significantly increased in net photosynthetic rate by 167.2%. In addition, when rice shoots were sprayed with Pro-Ca under salt stress, the Na^+^ concentration was considerably reduced by 17.1% compared to salt treatment. In conclusion, Pro-Ca regulates antioxidant mechanisms and photosynthesis to aid in the growth of rice seedlings under salt stress.

## Introduction

Salt stress is one factor limiting crop yield [1]. Previous studies have shown that salt stress leads to the accumulation of Na^+^ and the deficiency of Ca^2+^, K^+^ and Mg^2+^, then destroys the ion balance in plants, leading to osmotic stress and a water deficit, and finally reduces photosynthetic rates and inhibits plant growth [2]. In addition, salt stress will lead to a large accumulation of reactive oxygen species (ROS), thus causing oxidative stress to cause membrane peroxide and accelerating the degree of membrane damage. When crops suffer from salt stress, they can resist the damage caused by stress in many ways. When the ion balance is disturbed, plants will conduct permeation regulation through ion transport and add permeation regulation substances to maintain the ion balance of cells. Plants also require antioxidant defense mechanisms to manage ROS metabolism [3]. However, when crops cannot resist stress by themselves, they need external means to help them resist stress. It has been reported that using plant growth regulators can be regarded as a cost-effective and efficient technique for plants to attain equilibrium in plant systems under adverse situations [4].

A cyclohexane carboxylic acid called prohexadione-calcium (Pro-Ca) is used to control several developmental processes. Pro-Ca suppressed gibberellin (GA) production by preventing the 3, -hydroxylation of GA20 to GA1 [5]. Many studies have shown that Pro-Ca suppressed rice lodging under field conditions by blocking the GA biosynthesis pathway [6, 7]. In addition, Pro-Ca can improve the impacts of biotic and abiotic stress [6, 8], such as fungal, salt, and cold injury [9, 10]. According to research on the salt tolerance of broad beans and soybeans, exogenous Pro-Ca applications can regulate the levels of hormones, particularly gibberellin, abscisic acid, and cytokinin, as well as gas exchange and antioxidant enzyme activities in order to regulate photosynthetic efficiency in various plant species under salt stress [9, 11]. However, the effects and mechanisms by which Pro-Ca affects the tolerance of rice seedlings to salt stress are not well documented.

Rice (*Oryza sativa* L.), the primary crop, accounts for about 50% of the world’s food consumption. The rice seedling stage is the critical stage of rice morphogenesis which is also sensitive to salt stress. Due to limited reports on the protective effects of Pro-Ca on rice under normal and salt stress conditions, we here conducted to determine 1) whether Pro-Ca confers salt tolerance to rice seedlings; 2) if Pro-Ca played a protective role for salt-stressed rice, how the physiological parameters (photosynthetic characteristics, antioxidant system, and ion balance) can change, and whether differentially expressed genes (DEGs) could be enriched into these pathways. The physiological, transcriptome analysis and quantitative real-time PCR will be used to verify the hypothesis of this study, which may provide a theoretical reference for the application of Pro-Ca in salt stress.

## Materials and methods

### Experimental design

The experiment was carried out in 2020 at the College of Coastal Agricultural Sciences of Guangdong Ocean University, Zhanjiang, Guangdong Province, China (110.3°E, 21.1°N). Rice seeds were obtained from the College of Coastal Agricultural Sciences of Guangdong Ocean University. Rice variety IR29 was selected as the test material. The rice seeds of uniform size and color were selected, then sterilized the surface of the seeds with 5% NaClO solution first, and after three minutes washed them clean with distilled water, then sow the seeds on the 2kg plastic basin (19 cm × 14 cm × 17 cm) with holes at the bottom, each basin is filled with soil mixed with red brick soil and sand in a ratio of 3:1.

### Salt stress and prohexadione-calcium treatments

Salt stress and Pro-Ca treatment were carried out at the 1.5 leaf stage of rice. Then the pots were artificially divided into three groups, and each group had 30 pots:

1. CK (control): normal water
2. S: 50 mmol·L^−1^ NaCl salt solution
3. S + Pro-Ca: 50 mmol·L^−1^ NaCl salt solution + 100 mg·L^−1^ Pro-Ca.

The leaves in this study were sprayed with Pro-Ca (100 mg·L^−1^) once the desired saline concentration was achieved. The plants were grown in the sunlight greenhouse, with day/night temperatures of 26/22°C, day/night photoperiod of 10/14 h, and relative humidity of 70%. The experiment was designed according to a completely randomized block design.

### Morphological measurements

Samples were collected at the 6th, 12th, 24th hours. The plant height and stem diameter of each individual were measured with a ruler and vernier caliper. The shoots were dried for 30 min at 105°C and 72 h at 85°C; shoot dry weights were measured with an electronic analytical balance [9].

### Measurements of enzyme activity

Samples were collected at the 6th, 12th, 24th hours. The 500 mg of leaves were weighed and homogenized in 10 mL of phosphate buffer (50 mM pH 7.8), containing 1% polyvinylpyrrolidone (PVP), followed by centrifugation at 12000×g for 20 min at 4°C. The supernatant was collected to determine the activities of superoxide dismutase (SOD) [12], peroxidase (POD) [13], and ascorbate peroxidase (APX) [14].

### Measurements of membrane lipid peroxidation

Membrane lipid peroxidation level was evaluated by quantifying the content of malondialdehyde (MDA). MDA content was measured during the 2.5 leaf stage of the rice. Frozen leaf samples (500 mg) were homogenized in 10 mL 10% trichloroacetic acid (TCA) and centrifuged at 6000×g for 20 min. The supernatant (1 mL) was added to 2 mL of a reaction mixture containing 0.6% (v/v) thiobarbituric acid (TBA) and 10% (w/v) TCA. The mixture was boiled at 100°C for 15 min and centrifuged at 4000×g for 20 min after cooling. The supernatant was collected to determine the content of MDA [15].

### Measurements of Na^+^ content, K^+^ content and the ratio of K^+^/Na^+^

Na^+^ and K^+^ contents were measured during the 2.5 leaf stage of the rice. The rice shoots were washed with clean water and then dried at 75°C until attaining a constant weight. The sample was crushed and digested with HNO_3_ and HClO_4_ (4:1, v/v) and concentrated in a microwave oven (Mars, CEM Inc., New York, USA) [16]. The concentrations of Na^+^ and K^+^ were determined by atomic absorption spectrometry (PerkinElmer PinAAcle 900, Waltham Massachusetts, USA).

### Measurement of chlorophyll (Chl) content

Chlorophyll a (Chl a), chlorophyll b (Chl b), and carotenoids (Car) contents were measured at the 2.5 leaf stage of rice according to the method from Lichtenthaler [17]. In short, 0.1 g of fresh leaf tissue was soaked in 10 mL of 95% ethanol for 26 h at -4°C in the dark. The extract was used to determine pigment content by ultraviolet spectrophotometry.

### Measurement of gas-exchange parameters

Photosynthetic properties of functioning leaves, including net photosynthetic rate (Pn), stomatal conductance (Gs), and transpiration rate (Tr), were measured between 9:00 and 11:00 AM on bright, sunny days at the 2.5 leaf stage using an LI-6400XT (Li-COR 6400, Li-COR Inc., Nebraska, USA).

### RNA-seq analysis

RNA quantification and qualification: RNA concentration and purity was measured using NanoDrop 2000 (Thermo Fisher Scientific, Wilmington, DE). RNA integrity was assessed using the RNA Nano 6000 Assay Kit of the Agilent Bioanalyzer 2100 system (Agilent Technologies, CA, USA). Library preparation for Transcriptome sequencing: a total amount of 1 μg RNA per sample was used as input material for the RNA sample preparations. Sequencing libraries were generated using NEBNext UltraTM RNA Library Prep Kit for Illumina (NEB, USA) following manufacturer’s recommendations and index codes were added to attribute sequences to each sample. Briefly, mRNA was purified from total RNA using poly-T oligo-attached magnetic beads. Fragmentation was carried out using divalent cations under elevated temperature in NEBNext First Strand Synthesis Reaction Buffer (5X). First strand cDNA was synthesized using random hexamer primer and M-MuLV Reverse Transcriptase. Second strand cDNA synthesis was subsequently performed using DNA Polymerase I and RNase H. Remaining overhangs were converted into blunt ends via exonuclease/polymerase activities. After adenylation of 3’ ends of DNA fragments, NEBNext Adaptor with hairpin loop structure were ligated to prepare for hybridization. In order to select cDNA fragments of preferentially 240 bp in length, the library fragments were purified with AMPure XP system (Beckman Coulter, Beverly, USA). Then 3 μl USER Enzyme (NEB, USA) was used with size-selected, adaptor-ligated cDNA at 37°C for 15 min followed by 5 min at 95°C before PCR. Then PCR was performed with Phusion High-Fidelity DNA polymerase, Universal PCR primers and Index (X) Primer. At last, PCR products were purified (AMPure XP system) and library quality was assessed on the Agilent Bioanalyzer 2100 system. Clustering and sequencing: the clustering of the index-coded samples was performed on a cBot Cluster Generation System using TruSeq PE Cluster Kit v4-cBot-HS (Illumia) according to the manufacturer’s instructions. After cluster generation, the library preparations were sequenced on an Illumina platform and paired-end reads were generated.

#### Data analysis

The raw reads were further processed with a bioinformatic pipeline tool, BMK Cloud (www.biocloud.net) online platform. Raw data (raw reads) of fastq format were firstly processed through in-house perl scripts. In this step, clean data (clean reads) were obtained by removing reads containing adapter, reads containing ploy-N and low quality reads from raw data. At the same time, Q20, Q30, GC-content and sequence duplication level of the clean data were calculated. All the downstream analyses were based on clean data with high quality. Comparative analysis: the adaptor sequences and low-quality sequence reads were removed from the data sets. Raw sequences were transformed into clean reads after data processing. These clean reads were then mapped to the reference genome sequence. Only reads with a perfect match or one mismatch were further analyzed and annotated based on the reference genome. Hisat2 tools soft were used to map with reference genome. Gene functional annotation: gene function was annotated based on the following databases: Nr (NCBI non-redundant protein sequences); Nt (NCBI non-redundant nucleotide sequences); Pfam (Protein family); KOG/COG (Clusters of Orthologous Groups of proteins); Swiss-Prot (A manually annotated and reviewed protein sequence database); KO (KEGG Ortholog database); GO (Gene Ontology). Quantification of gene expression levels: quantification of gene expression levels Gene expression levels were estimated by fragments per kilobase of transcript per million fragments mapped. The formula is shown as follow: FPKM = (cDNA Fragments) / (Mapped Fragments (Millions)*Transcript Length(kb)). The RNA sequence data reported in this paper have been deposited in the Genome Warehouse in National Genomics Data Center, Beijing Institute of Genomics, Chinese Academy of Sciences / China National Center for Bioinformation, under accession number PRJCA015728 that is publicly accessible at https://ngdc.cncb.ac.cn/gwh.

### Differential expression analysis

For the samples with biological replicates: differential expression analysis of two conditions/groups was performed using the edgeR. edgeR provides statistical routines for determining differential expression in digital gene expression data using a model based on the negative binomial distribution. The Q-Value < 0.01 & Fold Change ≥1.5 was set as the threshold for significantly differential expression. Gene Ontology (GO) enrichment analysis of the differentially expressed genes (DEGs) was implemented by the GOseq R packages based Wallenius non-central hyper-geometric distribution [18], which can adjust for gene length bias in DEGs. KEGG [19] is a database resource for understanding high-level functions and utilities of the biological system, such as the cell, the organism and the ecosystem, from molecular-level information, especially large-scale molecular datasets generated by genome sequencing and other high-throughput experimental technologies (http://www.genome.jp/kegg/). We used KOBAS [20] software to test the statistical enrichment of differential expression genes in KEGG pathways.

### Gene expression qRT-PCR validation

To verify the accuracy of transcriptome data, we performed qRT-PCR on the corresponding DEGs in rice samples at different time points of 6th, 12th and 24th hours. Real-time qRT-PCR analysis was conducted to examine the expression patterns of S and S + Pro-Ca samples. A total of 5 candidate genes were selected and used to validate the transcriptome data using the same RNA samples of RNA-sequencing. The total RNA in the sample was extracted by Trizol method. Operated as the handbook of TUREscript 1st Stand cDNA SYNTHESIS Kit to systhesis cDNA. Primers were disigned by Beacon Designer 7.9. The list of primers used for the qRT-PCR is shown in S2 Table. Quantitative real-time PCR (qRT-PCR) was then performed. The qRT-PCR cycles had the following reaction conditions: Step1: 95℃-3 min; Step2: 95°C -10 s; Step3: 58°C -30 s + plate read; Step5: Go to step2, 39 cycles; Step6: Melt curve analysis (60°C ~ 95°C, +1°C/cycle, holding time 4 s). After add all components, centrifuge at 6,000 rpm for 1 minutes to keep all components in the bottom. *UBQ10* (Accession: AK101547) was used as the internal reference gene and as an internal control to normalize the expression levels [21]. Each PCR was repeated three times.

### Data analysis

SPSS 20.0 (IBM Inc., Chicago, IL, USA) was used for variance analysis of all data. Duncan’s multiple range test and independent sample T-test (P < 0.05) was used to discover variations between means. Figure preparation was performed via Origin Pro 9.1 (Origin Lab, Northampton, MA, USA).

## Results

### Pro-Ca promoted rice seedlings’ development under salt stress

**Fig 1:**
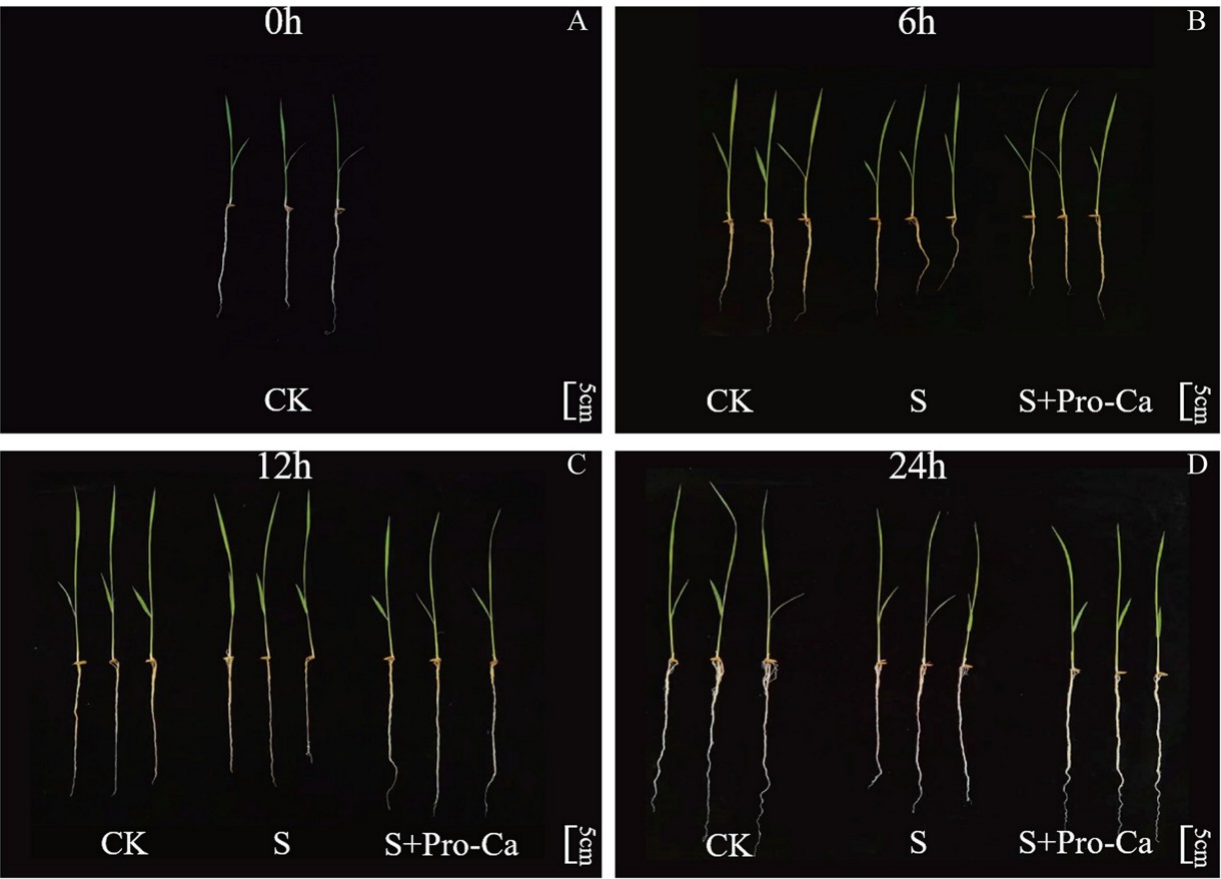
The effect of Pro-Ca on rice seedlings’ phenotype under salt stress. The phenotype at 0h(A), 6h(B), 12h(C), and 24h(D). Abbreviations: CK, control (normal water); S, salt stress; and S + Pro-Ca, salt stress plus foliar Pro-Ca application. Scale bar = 5cm.

As shown in Fig 1A, there was no significant difference in the phenotype of rice seedlings without treatment (0h). However, salt stress gradually destroys rice seedlings with the extension of salt stress time. Especially when the rice seedlings were stressed by salt for 24th hours, the rice suffered the most serious damage. As shown in Fig 1D, the rice seedlings were damaged by salt stress, with seedlings showing the most prominent symptoms of toxicity, such as short roots. However, when Pro-Ca was applied to the rice seedlings, they had thicker stalks than those subject to salt treatment only.

**Fig 2:**
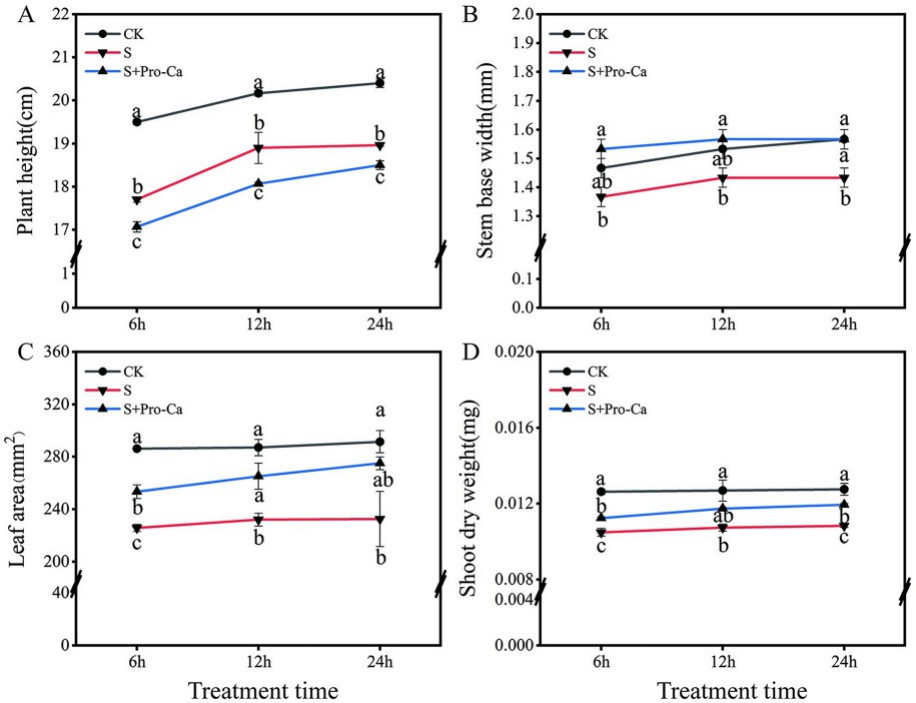
The effect of Pro-Ca on rice seedlings morphology under salt stress. Plant height (A), stem base width (B), leaf area (C), and shoot dry weight (D) under no stress and salt stress (50 mmol·L^−1^) with or without Pro-Ca (100 mg·L^−1^) spray. Abbreviations: CK, control (normal water); S, salt stress; and S + Pro-Ca, salt stress plus foliar Pro-Ca application. Values represent mean ± SE (n = 3), and different lowercase letters indicate significant differences according to Duncan’s test.

Compared with normal growth conditions, salt treatment inhibited the growth of rice seedlings. As shown in Fig 2, compared to normal growth conditions, salt treatment significantly reduced stem base width by 8.5% at the 24th hour (Fig 2B); salt treatment reduced the shoot dry weight by 16.9%, 15.4%, and 15.2% from the 6th to 24th hour (Fig 2D). During 6th to 24th hour, salt treatment dramatically decreased plant height and leaf area by 9.2–7.0% and 21.1–20.2%, respectively (Fig 2A and 2C). Pro-Ca alleviated the inhibitory effect of salt stress on the morphology of rice seedlings (Fig 2). The application of exogenous Pro-Ca under salt stress significantly increased the stem base width by 12.2%, 9.3%, and 9.3% from the 6th to 24th hour compared to the salt treatment (Fig 2B). In addition, exogenous spraying Pro-Ca under salt stress significantly increased the leaf area by 12.2% and 14.3% at the 6th and 12th hours compared to the saline treatment (Fig 2C). Meanwhile, exogenous Pro-Ca under salt stress treatment shoot dry weight was significantly increased by 7.2% and 10.4% at the 6th and 24th hours compared to the saline treatment, respectively (Fig 2D). Exogenous Pro-Ca can reduce the inhibitory effect of salt stress on rice growth, which is manifested in the increase of plant height, the thickening of stem base width, and the increase of leaf area and dry weight.

### Pro-Ca regulated K^+^/Na^+^ homeostasis to protect rice seedlings from salt stress

**Fig 3:**
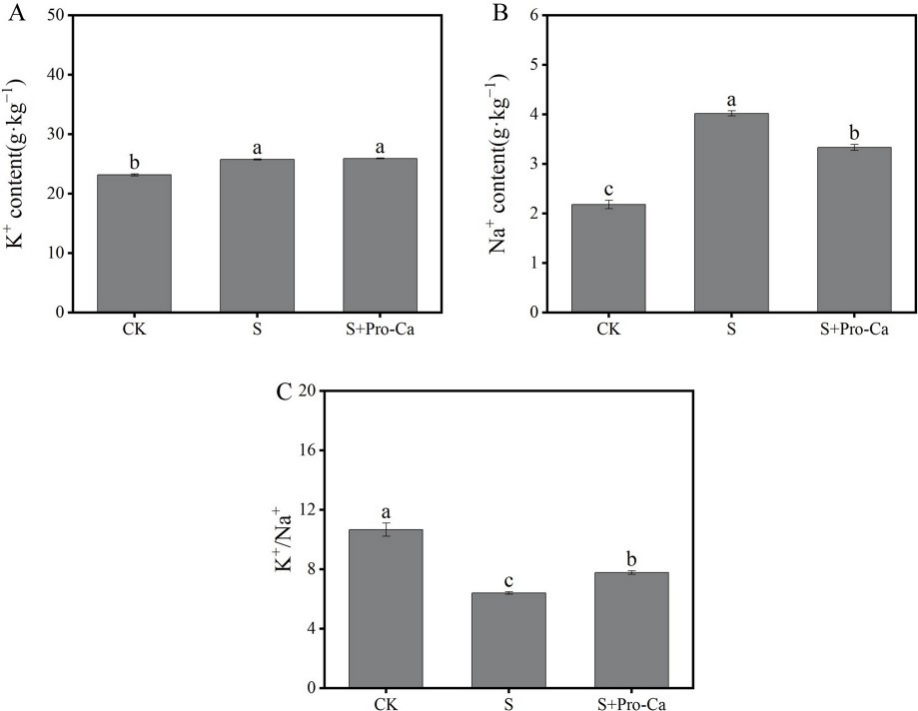
Exogenous prohexadione-calcium (Pro-Ca) application conferred salt stress resistance of rice seedlings. K^+^ content (A), Na^+^ content (B), and K^+^/Na^+^(C) under no stress and salt stress (50 mmol·L^−1^) with or without Pro-Ca (100 mg·L^−1^) spraying. Abbreviations: CK, control (normal water); S, salt stress; and S + Pro-Ca, salt stress plus foliar Pro-Ca application. Values represent mean ± SE (n = 3), and different lowercase letters indicate significant differences according to Duncan’s test.

Compared with normal growth conditions, salt stress significantly increased the content of Na^+^ by 84.4% and decreased the ratio of K^+^/Na^+^ by 39.9% in rice shoots (Fig 3B and 3C). Exogenous Pro-Ca under salt stress significantly decreased the Na^+^ content in rice shoots by 17.1% compared to salt treatment (Fig 3B). In contrast, exogenous Pro-Ca under salt stress significantly increased the K^+^ content in rice shoots compared to salt treatment (Fig 3A). In addition, exogenous Pro-Ca also significantly inhibited decreases in the K^+^: Na^+^ ratio due to salt stress (Fig 3C).

### Analysis of DEGs under Pro-Ca treatment in response to salt stress

The Q30 value was over 93.99% in all samples, and the GC content was 50.67%. The genome map rate accounted for more than 83.15%, and the unique mapped reads rate was 80.54% to 83.45% (Table 1). It has been proved that the base recognition degree is high quality and reliable, the reference genome selection is appropriate, and the comparison effect is better.

**Table 1:** Summary of sequence data.

Sample	Treatment time	Clean reads	Q30 (%)	GC (%)	Mapped reads rate (%)	Uniq mapped Reads rate (%)	Multiple Map Reads rate (%)
**CK**	0h	24760181	94.25%	51.94%	83.22%	80.54%	2.68%
**S**	6h	25642908	94.38%	52.46%	85.40%	83.34%	2.05%
	12h	24856624	94.07%	50.67%	84.89%	82.76%	2.13%
	24h	29208804	94.28%	52.45%	83.15%	81.15%	2.00%
**S+Pro-Ca**	6h	24624117	94.23%	52.00%	85.53%	83.45%	2.09%
	12h	25516281	93.99%	50.67%	84.82%	82.68%	2.15%
	24h	25755349	94.16%	52.28%	83.34%	81.33%	2.00%

Abbreviations: CK, control (normal water); S, salt stress; and S + Pro-Ca, salt stress plus foliar Pro-Ca application.

**Fig 4:**
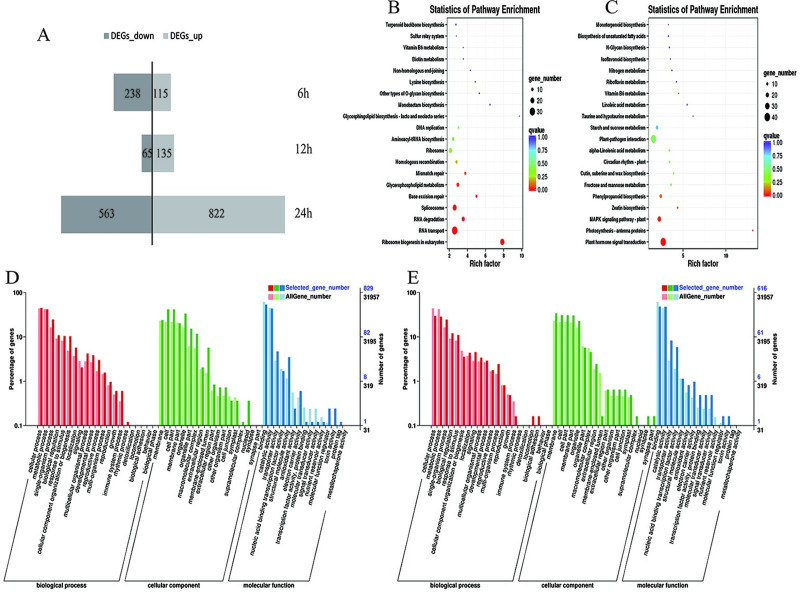
Analysis of DEGs under Pro-Ca treatment in response to salt stress. Numbers of DEGs (A), KEGG enrichment of up-regulated (B) and down-regulated (C) DEGs in S vs. S + Pro-Ca at the same treatments times, and Summary of Gene Ontology (GO) categories of the up-regulated (D) and down-regulated (E) DEGs in S vs. S + Pro-Ca at the same treatment times, respectively. Abbreviations: CK, control (normal water); S, salt stress; and S + Pro-Ca, salt stress plus foliar Pro-Ca application.

To understand the differences between salt treatment with Pro-Ca treatment, gene expression profiles in S and S + Pro-Ca treatments were further analyzed through FPKM values. Q-Value<0.01 and |FC|>1.5 were used as the screening criteria for differential genes. There were 353, 200, and 1385 DEGs were identified at the 6th, 12th, and 24th hours, respectively, of which a total of was identified 115 up-regulated genes and 238 down-regulated genes (6th hour), 135 up-regulated genes, and 65 down-regulated genes (12th hour), and 822 up-regulated and 563 down-regulated genes (24th hour), which may be associated with Pro-Ca enhancing salt tolerance in rice (Fig 4A).

GO analyses were performed to clarify the response to salt stress and Pro-Ca. In the comparative analysis of DEGs of S vs S + Pro-Ca, the DEGs of these GO terms were classified into ‘cellular process’, ‘metabolic process’, and ‘single-organism process’ in the biological process; ‘cell’, ‘cell part’, and ‘membrane’ in the cell components; ‘binding’, ‘catalytic activity’, and ‘transporter activity’ were in the molecular function (Fig 4D and 4E). In addition, 40 standard metabolic processes were most enriched during the process of Pro-Ca treatment to elucidate the metabolic pathways involved under salt stress. Among these top pathways, RNA transport, ribosome biogenesis in eukaryotic, spliceosome, RNA degradation, and base excision repair were associated with many up-regulated genes (Fig 4B). Furthermore, among these top pathways, the plant hormone signal transduction, MAPK signaling pathway-plant, photosynthesis-antenna proteins, zeatin biosynthesis, and phenylpropanoid biosynthesis were associated with many down-regulated genes (Fig 4C). In brief, these genes related to metabolic pathways may be involved in regulating Pro-Ca in rice seedlings under salt stress.

### Pro-Ca regulated photosynthesis, chlorophyll metabolism, and related gene expression to protect rice seedlings from salt stress

**Fig 5:**
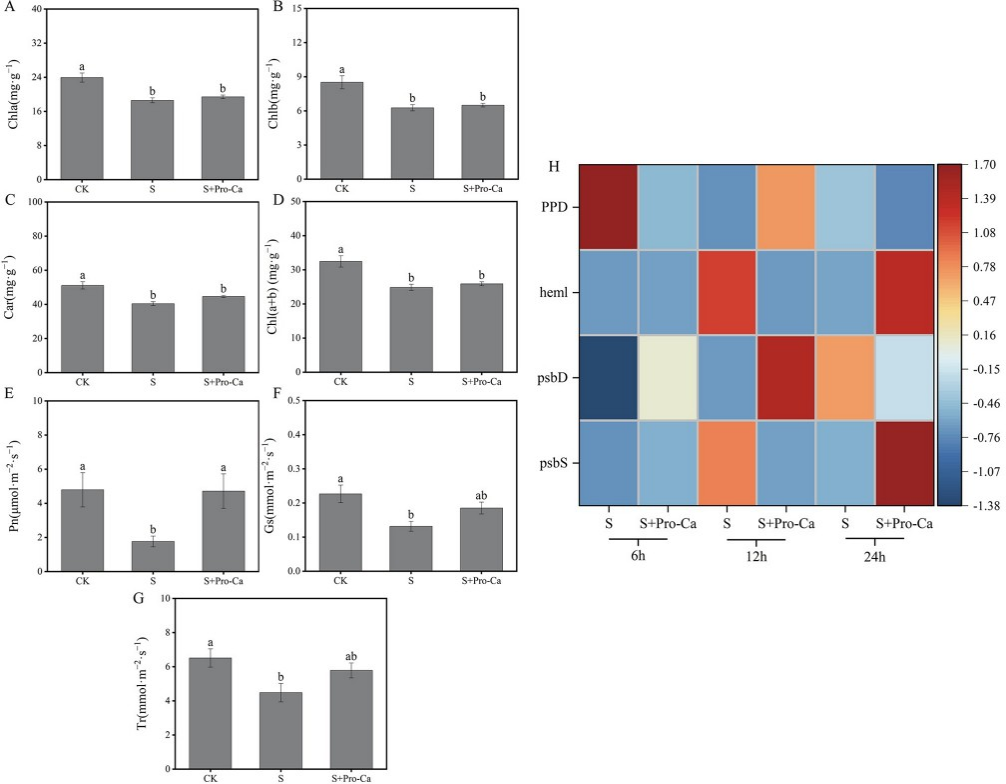
Exogenous prohexadione-calcium (Pro-Ca) application conferred salt stress resistance of rice seedlings. Chl a content (A), Chl b content (B), Car content (C), total chlorophyll content (D), net photosynthetic rate (E), stomatal conductance (F), transpiration rate (G), and photosynthesis and chlorophyll metabolism-related gene (H) under no stress and salt stress (50 mmol·L^−1^) with or without Pro-Ca (100 mg·L^−1^) spraying. The expression level changes of genes in each group were described as log2 fold change of FPKM. Abbreviations: CK, control (normal water); S, salt stress; and S + Pro-Ca, salt stress plus foliar Pro-Ca application. Values represent mean ± SE (n = 3), and different lowercase letters indicate significant differences according to Duncan’s test.

Salt stress inhibited chlorophyll synthesis and decreased Car content in rice leaves. Similarly, Chl a and Chl b, and total chlorophyll contents under salt stress were observed to decrease compared to normal growth conditions. Pro-Ca increased Chl a, Chl b, total chlorophyll, and Car contents compared to salt stress treatment at the 2.5 leaf stage compared to salt stress treatment. Compared with salt stress treatment, spraying Pro-Ca under salt stress increased Chl a, Chl b, total chlorophyll, and Car contents by 4.4%, 3.8%, 4.3% and 10.5%. Compared to normal growth conditions, plants treated only with salt stress displayed significant decreases in net photosynthetic rate (Pn), stomatal conductance (Gs), and transpiration rate (Tr) at the 2.5 leaf stage (Fig 5A–5D). In contrast, compared to salt stress treatment, the application of Pro-Ca under salt stress alleviated the decline in gas exchange, as indicated by a significant increase of Pn by 167.2%, an increase of Gs by 40.8%, and an significant increase of Tr by 29% (Fig 5E–5G).

From the transcriptome analysis, we found that Pro-Ca regulated the expression of photosynthesis, porphyrin and chlorophyll metabolism-related genes (Fig 5H). Between S and S + Pro-Ca, there were 4 DEGs. Compared with salt treatment, photosystem Ⅱ 22kDa protein gene (*psbS*) was up-regulated after Pro-Ca treatment at the 6th hour. Moreover, one photosystem Ⅱ P680 reaction center D2 protein gene (*psbD*) was up-regulated by Pro-Ca under salt stress. Meanwhile, glutamate-1-semialdehyde 2,1-aminomutase gene (*heml*) was up-regulated after Pro-Ca treatment compared to salt treatment. In addition, compared to salt treatment, pheophorbidase (*PPD*) gene was down-regulated after Pro-Ca treatment. These genes were assigned to the porphyrin and chlorophyll metabolism (ko00860) or photosynthesis (ko00195). Meanwhile, *psbD* was assigned to photosynthetic electron transport in photosystem II (GO:0009772) (S1 Table). These studies show that Pro-Ca under salt stress can increase photosynthetic pigment content in rice seedlings by regulating photosynthesis and chlorophyll synthesis-related genes and promoting rice photosynthesis.

### Pro-Ca regulated antioxidant enzyme activities, redox-related gene expression, and reduced MDA content to protect rice seedlings from salt stress

**Fig 6:**
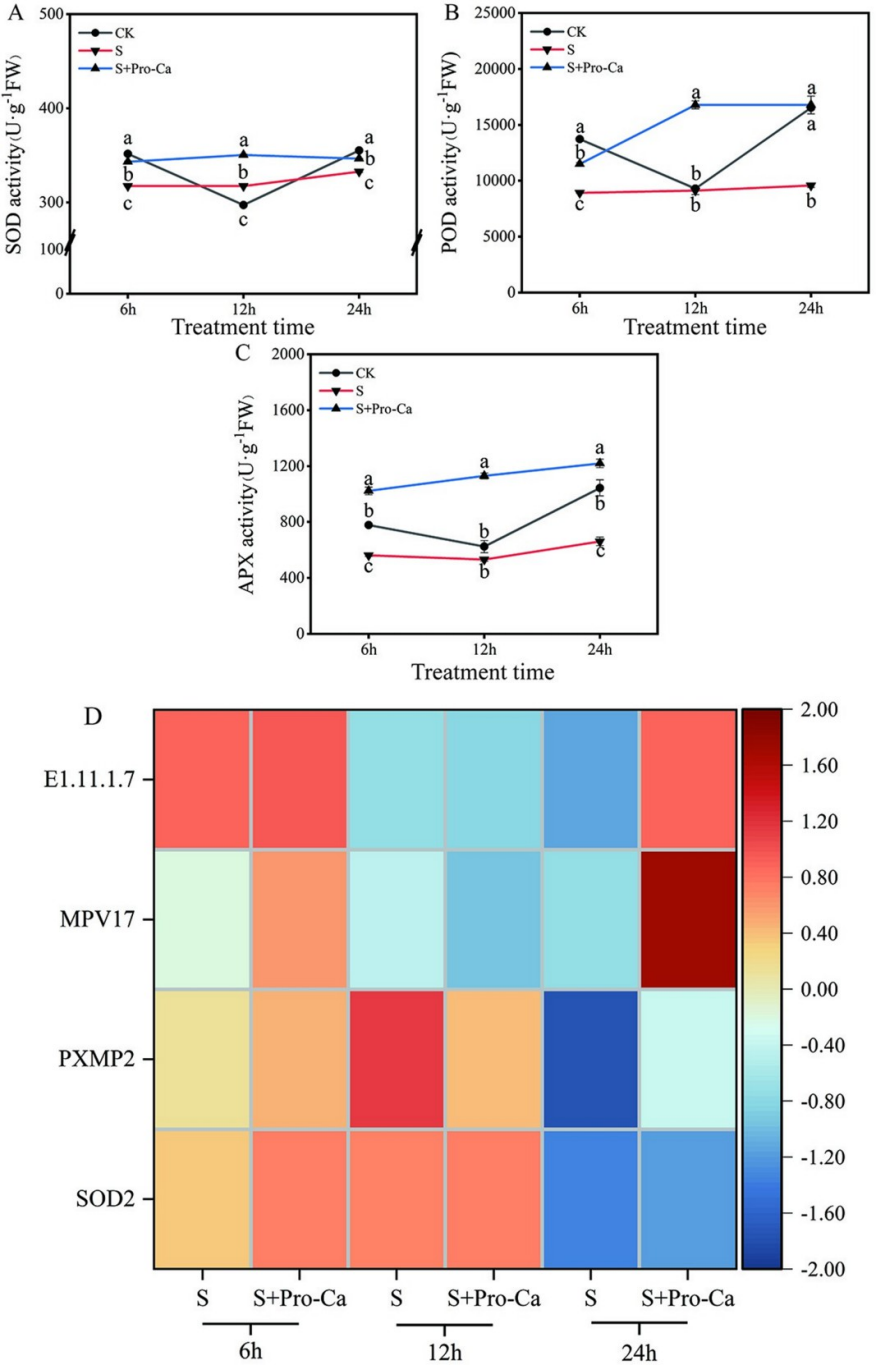
Exogenous prohexadione-calcium (Pro-Ca) application conferred salt stress resistance of rice seedlings. SOD (A), POD (B), APX (C) activity content and antioxidant enzyme activities-related gene (D) under no stress and salt stress (50 mmol·L^−1^) with or without Pro-Ca (100 mg·L^−1^) spraying. The expression level changes of genes in each group were described as log2 fold change of FPKM. Abbreviations: CK, control (normal water); S, salt stress; and S + Pro-Ca, salt stress plus foliar Pro-Ca application. Values represent mean ± SE (n = 3), and different lowercase letters indicate significant differences according to Duncan’s test.

Compared with the control, salt treatment reduced APX activity by 27.6% and 36.6% at the 6th and 24th hours; reduced POD activity by 35.1% and 42.1% at the 6th and 24th hours; in addition, reduced SOD activity by 9.7% and 6.3% at the 6th and 24th hours (Fig 6A–6C). Under salt stress conditions, the exogenous Pro-Ca mitigated the overall effect of salt stress. Compared to salt treatment, exogenous Pro-Ca under salt stress resulted in a significant increase in APX activity by 81.7%, 112.1%, and 84.2% from the 6th to 24th hours (Fig 6C), POD activity by 29%, 84.2%, and 75.2% from 6th to 24th hours (Fig 6B), and SOD activity by 8.2% - 3.5% from 6th to 24th hours compared to the salt treatment (Fig 6A). These results indicated that exogenous Pro-Ca might alleviate the effects of salt stress by increasing antioxidant enzyme activity.

Antioxidant enzymes can protect plants against oxidative damage induced by stress. Antioxidant enzyme-related genes were detected, including peroxidase and superoxide dismutase. Between S and S + Pro-Ca, four genes were regulated. Compared with the salt treatment, the superoxide dismutase, Fe-Mn family gene (*SOD2*) was up-regulated after Pro-Ca treatment on the 24th hour. The peroxisomal membrane protein 2 (*PXMP2*), protein *Mpv17* gene (*MPV17*), and the peroxidase geng (*E1*.*11*.*1*.*7*) were up-regulated after Pro-Ca treatment compared to the salt treatment on the 24th hour (Fig 6D). In addition, their genes (*SOD2*, *PXMP2*, and *MPV17*) were assigned to the peroxisome pathway (ko04146), one gene (*SOD2*) was assigned to the superoxide dismutase activity (GO:0004784), and one gene (*E1*.*11*.*1*.*7*) was peroxidase activity (GO:0004601), response to oxidative stress (GO:0006979), and hydrogen peroxide catabolic process (GO:0042744) (S1 Table).

In order to verify the effect of Pro-Ca on the antioxidant process of rice under salt stress, we determined the MDA content (S1 Fig). Compared with the control, salt treatment significantly increased MDA content by 5.8%. Under salt stress conditions, the exogenous Pro-Ca mitigated the overall effect of salt stress. Compared to salt treatment, exogenous Pro-Ca under salt stress resulted in a significant decrease in MDA content by 5.8%. These results indicated that exogenous Pro-Ca might alleviate the membrane lipid peroxidation damage of rice under salt stress.

### Validation of key differentially expressed genes

**Fig 7:**
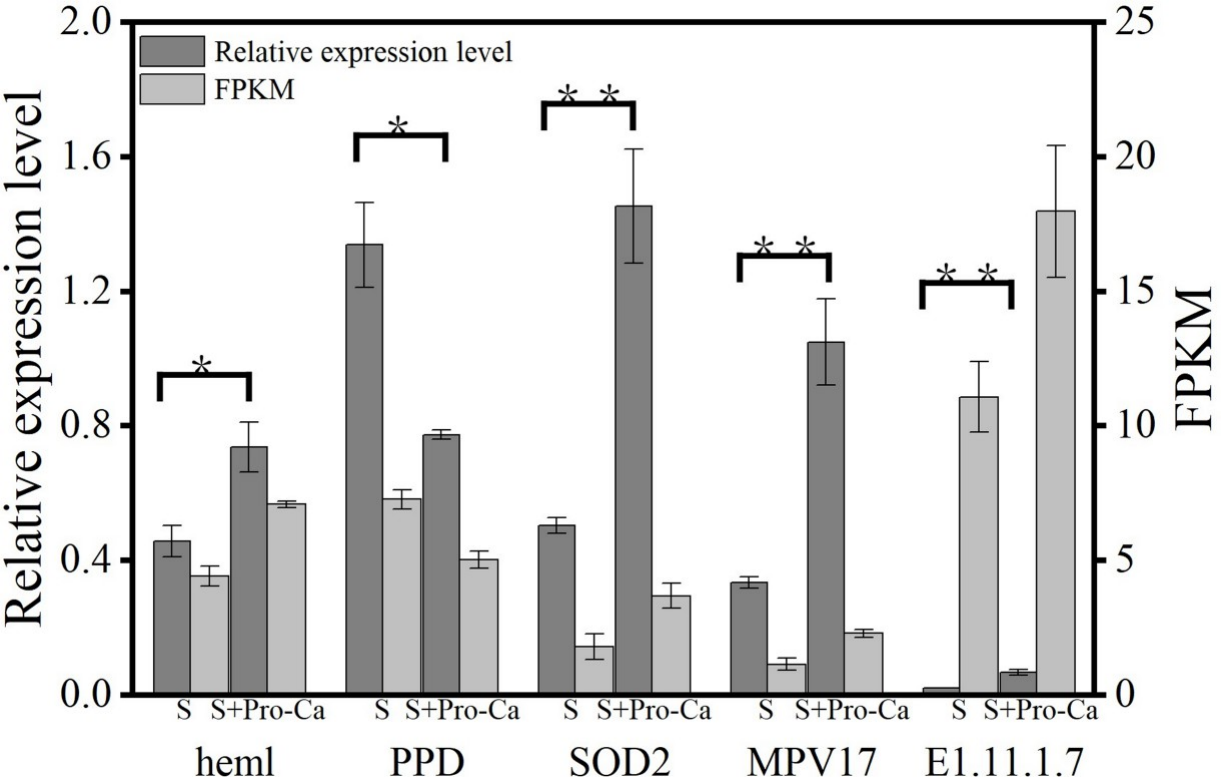
Validation of key differentially expressed genes. Relative expression level: The relative expression level of 5 genes obtained by quantitative real-time PCR (qRT-PCR) analysis; FPKM: The expression level of 5 genes is based on the fragments per kilobase of transcripts per million mapped fragments (FPKM) value. The error bars represent the SE from three replicates. Abbreviations: S, salt stress; and S + Pro-Ca, salt stress plus foliar Pro-Ca application. Values represent mean ± SE (n = 3); *: P<0.05 (Relative expression level); **: P<0.01 (Relative expression level).

To verify the accuracy of transcriptome data, five differential genes (*PPD* in rice after 6h treatment, *heml* in rice after 12h treatment, *SOD2* in rice after 24h treatment, *MPV17* in rice after 24h treatment, *E1*.*11*.*1*.*7* in rice after 24h treatment) involved in Pro Ca regulation at different times were screened for qRT-PCR expression difference verification (Fig 7). The analysis showed that the statistical results of qRT-PCR of five different genes were consistent with the results of RNA seq sequencing. The trends of up-regulation and down-regulation were consistent, but the specific expression differences varied with different experimental methods. The results of qRT-PCR validation showed that: (1) the expression of photosynthesis, porphyrin, and chlorophyll metabolism-related genes *heml* was up-regulated, while the expression of gene *PPD* was down-regulated; (2) The expression of antioxidant enzyme-related genes *SOD2*, *MPV17*, *E1*.*11*.*1*.*7* was up-regulated. Therefore, RNA seq data can be used for in-depth research on genes related to Pro Ca alleviating salt stress in rice.

## Discussion

Plant morphological changes under salt stress include leaf rolling, chlorosis, reduced biomass, and shorter plant height, ultimately leading to inhibitions in the growth and development of plants, and even leading to reductions in grain yield [22]. Our results show that salt treatment significantly reduced the plant height, stem base width, leaf area, and shoot dry weight (Fig 2). Results of the current study further confirmed the negative effect of salt stress treatments on rice growth. In contrast, the exogenous application of Pro-Ca significantly enhanced the stem base width, leaf area, and shoot dry weight but decreased the plant height. These results were similar to previous studies [7, 9]. They reported that Pro-Ca decreased plant height and enhanced the stem base width by blocking the GA biosynthesis pathway to resist stress and prevent lodging.

Salt stress leads to physiological dehydration and osmotic stress in plants and has ion-toxicity effects on plants, causing Na^+^ accumulation in plant cells [23]. Na^+^ reduces enzyme activity, which has an adverse impact on the Calvin cycle and other metabolic processes [24]. Similar to other investigations, the present study found that salt stress increased the Na^+^ concentration of rice. In contrast, when exogenous Pro-Ca was applied to the rice leaves, the Na^+^ content decreased, and K^+^ content increased, thus increasing the K^+^/Na^+^ ratio (Fig 3). Previous studies have observed that excess Na^+^ in the cytoplasm also interferes with the uptake and transport of K^+^, causes K^+^/Na^+^ ratio imbalance, ultimately resulting in premature leaf senescence and even plant death [25, 26]. The possible explanation for our study’s phenomenon is that Pro-Ca enhances K^+^ absorption by promoting Na^+^ efflux, and promotes the decrease of the K^+^/Na^+^ ratio, ultimately reducing the ionic toxicity caused by salt stress. Therefore, our results show that Pro-Ca treatment had greater tolerance under saline conditions than without Pro-Ca treatment and that this was associated with the maintenance of lower Na^+^ concentrations in the shoots of Pro-Ca treatment.

Dry matter accumulation depends on photosynthesis to fix assimilates. Excess ROS produced under stress will damage the photosynthetic structure of plants, eventually leading to the decline of leaf net photosynthetic rate (Pn) and the weakening of CO_2_ fixation capacity [27]. Our results show that salt treatment significantly reduced the shoot’s dry weight. At the same time, stress considerably inhibited the net photosynthetic rate (Pn) (Fig 5). These results were similar to previous studies [28]. They reported that salt stress inhibited the absorption of light energy by plants, promoted the senescence of plant seedlings, and finally reduced dry matter weight. Chlorophyll and carotenoids are essential photosynthetic pigments, and chlorophyll levels may drop when plants are exposed to salt stress [29]. Salt stress limits chlorophyll a/b synthesis, light energy absorption, photosystem I (PSI) and photosystem II (PSII) transmission and conversion, and reduces photosynthetic rate [30], therefore, lower chlorophyll content and damage to the chloroplast structure also causes the decrease of Pn [31]. In addition, the decrease in Pn can be due to the decline in stomatal conductance (Gs) [32]. These findings are similar to those of our study. In contrast, compared to the salt treatment, Pro-Ca significantly increased the gas exchange parameters (such as Pn, Gs) and the content of Chl a, Chl b, and Car. These results showed that using Pro-Ca promoted chlorophyll biosynthesis, protected chloroplast structure, and increased Pn. These results were similar to those reported by previous studies [9].

Salt stress will not only destroy ion balance and generate ion toxicity but also cause oxidative damage caused by excessive accumulation of ROS [33]. In rice, salt stress can also cause alterations in lipid peroxidation and antioxidant enzyme activity [34]. In the present study, the activities of antioxidant enzymes such as POD and APX were significantly reduced under salt stress compared to the control. However, while the SOD activity drastically increased at 12th hour under salt stress compared to the control, with the extension of salt stress time, SOD activity had a significant reduction at 24th hour under salt stress compared to the control (Fig 6). Our results show that the plant has the potential antioxidant defense mechanism which was activated to resist salt stress. Still, long-term continuous salt stress may destroy this defense mechanism, resulting in serious oxidative damage. Previous research has demonstrated that increasing SOD, POD, and APX activity was required to improve soybean saline-alkali tolerance [2]. SOD catalyzes the conversion of O_2_^∙−^ into O_2_ and H_2_O_2_, and further into nontoxic H_2_O and O_2_ [35]. APX plays an important role in detoxification and decomposition of H_2_O_2_, and is a synergistic enzyme with scavenging effect [36]. POD can catalyze the oxidation of substrates by using H_2_O_2_ as an electron acceptor [37]. Therefore, antioxidant enzymes play an important part in the process of scavenging reactive oxygen species. Many studies have shown that Pro-Ca can promote ROS clearance in plants and alleviate oxidative damage caused by stress to plants. Similar to Bekheta et al. [11], our investigation found that plants treated with Pro-Ca showed increased SOD, POD, and APX activities (Fig 6). Pro-Ca reduced oxidative damage caused by salt stress possibly by directly increasing antioxidative enzyme activity [9]. In addition, in this study, salt stress significantly increased the MDA content in rice, but Pro-Ca inhibited the increase of MDA content in rice caused by salt stress (S1 Fig). MDA is an oxidation product produced by reactive oxygen species attacking plants, which can be used as an index to measure the degree of oxidative damage. Therefore, this result further proves the positive role of Pro-Ca in alleviating oxidative damage caused by salt stress. Therefore, we speculate that exogenous Pro-Ca increased the resistance of rice to salt stress by increasing antioxidative enzyme activities and reducing the reactive oxygen species damage.

In our study, S+Pro-Ca treatment exhibits higher stress resistance than S treatment; presumably, Pro-Ca promotes rice seedling’s development under salt stress by regulating antioxidant processes and photosynthesis. To further explore the mechanism of Pro Ca relieving salt stress in rice, we used RNA-sequencing analysis to analyze the transcriptional variations between S treatment and S+Pro-Ca treatment. Using the transcriptome analysis profile, there were 353, 200, and 1385 DEGs were identified at the 6th, 12th, and 24th hours that showed at least a 1.5 fold change in S+Pro-Ca treatment compared to S treatment, respectively, of which a total of was identified 115 up-regulated genes and 238 down-regulated genes (6th hour), 135 up-regulated genes, and 65 down-regulated genes (12th hour), and 822 up-regulated and 563 down-regulated genes (24th hour), which may be associated with Pro-Ca enhancing salt tolerance in rice (Fig 4A). Then we screened DEGs related to the antioxidant system and photosynthesis for further analysis.

We screened some important photosynthesis-related genes in the S+Pro-Ca treatment, which were expressed differently than the S treatment, resulting in high Pn in rice under salt stress. In this study, compared to the salt treatment, Pro-Ca significantly increased the gas exchange parameters (such as Pn, Gs, and Tr) and Chl a, Chl b, total chlorophyll, and Car contents (Fig 5). Therefore, we speculated that some important porphyrin and chlorophyll metabolism-related genes in S+Pro-Ca treatment are expressed differently compared to S treatment, resulting in high chlorophyll content in rice under salt stress. For example, *heml*, a glutamate-1-semialdehude 2,1-aminomutase gene, participates in chlorophyll biosynthesis and is the second step of chlorophyll biosynthesis, catalyzing L-glutamic acid -1-semi-aldehyde to generate a-aminolevulinic acid. Therefore, the up-regulation of *heml* observed in our study may promote chlorophyll biosynthesis, resulting in a higher chlorophyll content in S+Pro-Ca treatment. In addition, the *PPD*, as pheophorbidase, participates in the degradation of chlorophyll; the loss of function of this gene can prevent the degradation of chlorophyll [38], which was one of the causes of the high chlorophyll content. This study’s *PPD* was down-regulated under Pro-Ca treatment compared to the salt treatment (Fig 5H). The results showed that Pro-Ca alleviated the reduction of chlorophyll content by down-regulating *PPD* expression under salt stress. Photosystem Ⅱ (PSⅡ) is an essential place for plants to perform an initial photosynthesis reaction on a capsule-like membrane. It can quickly respond to changes in the external environment and form a protection mechanism. Among them, *PsbS*, 22kDa protein encoded by the nuclear gene, as one of the protein components of PSⅡ super complex [39], is an indispensable part of dissipating excess light energy and plays an essential role in NPQ of chlorophyll fluorescence [40]. Many studies have shown that the lack of *PsbS* gene inhibits plant growth, and the increase in the expression level of this gene promotes plant growth and promotes the photosynthetic rate of plants [41–43]; therefore, the increase in photosynthesis can be attributed to the rise in the increase in the expression level of *PsbS* as evidenced by the simultaneous increase of Pn and *PsbS* expression levels in S+Pro-Ca treatment. Furthermore, PSⅡ contains two light capture complexes and a light reaction center. The light reaction center pigment of PSⅡ is called P680, while the pigment protein complex of the reaction center contains D2 protein. Previous studies have shown that under external stress, the expression of D2 protein (*PsbD*) in the PSⅡ P680 reaction center decreases, while the increase of *PsbD* expression can promote the self-repair of plants [44]. In this study, Pro-Ca treatment increased the expression levels of *PsbD* genes. The findings suggest that Pro-Ca alleviated salt stress by regulating the expression of *PsbD* in rice seedlings, thus promoting the photosynthesis process of rice seedlings under salt stress. Pro-Ca alleviated salt stress by regulating the expression of genes related to chlorophyll metabolism and photosynthesis in rice seedlings, thus increasing the chlorophyll content and promoting the photosynthesis process of rice seedlings under salt stress.

In addition, we speculated that some important antioxidant enzyme-related genes in the S+Pro-Ca treatment might be expressed differently compared to the S treatment, resulting in high salt resistance in rice. In this study, four antioxidant enzyme-related genes were up-regulated, as shown in Fig 6D. For example, *SOD2*, a superoxide dismutase Fe-Mn family gene, up-regulated in S+ Pro-Ca treatment. SOD is the antioxidant system’s initial line of defense and the backbone of the enzyme protection system [45]. Fe-SOD, Mn-SOD (*SOD2*), and Cu/Zn-SOD are the three types of SOD. Many studies have found that overexpression of the *SOD2* improved transgenic plants’ tolerance to different oxygen stressors. For example, Wang et al. transferred the *SOD2* gene from pea to rice, and the transgenic rice’s SOD activity was much greater than that of the control, enhancing the transgenic lines’ drought resistance [46]. Under salt treatment, overexpression of *SOD2* in rice increased SOD activity while also improving rice salt tolerance [47]. Therefore, in the current study, the increase in SOD activity can be attributed to the increase in up-regulation of *SOD2* gene expression, as evidenced by the simultaneous increase of SOD activity and *SOD2* gene expression in rice seedlings. Simultaneously, *E1*.*11*.*1*.*7* gene was up-regulated in S+ Pro-Ca treatment. *E1*.*11*.*1*.*7* gene is the peroxidase-related gene. Peroxidase is one of the redox enzymes and the marker enzyme of peroxidase. Participating in the antioxidant process of plants is the key to improving the stress tolerance of plants [48]. POD activity can be attributed to the increase in up-regulation of *E1*.*11*.*1*.*7* gene expression, as evidenced by the simultaneous increase of POD activity and *E1*.*11*.*1*.*7* gene expression in the rice seedlings. The findings suggest that Pro-Ca can increase rice SOD and POD activity by modulating the *SOD2* gene and *E1*.*11*.*1*.*7* gene. In addition, two ROS metabolic process-related genes, *PXMP2* and *MPV17*, were up-regulated in S+Pro-Ca treatment. The *PXMP2* gene, a peroxisomal membrane protein two genes, compared to the salt treatment, was also up-regulated in S+ Pro-Ca treatment. Peroxidase can produce and remove H_2_O_2_. *PXMP2*, as a widely expressed and rich trimer peroxidase membrane protein, participates in the metabolic process of ROS [49]. *MPV17* gene encodes a mitochondrial inner membrane protein, which can reduce the production of malondialondialects caused by oxidative damage under stress conditions and improve the tolerance of osmotic stress [50]. The present research showed that the loss of function of these genes destroyed the ROS metabolic process of the plant. In rice, excessive accumulation of ROS was a primary cause of low salt resistance. The findings suggest that the up-regulation of *PXMP2* and *MPV17* expression promotes ROS metabolism. As a result, Pro-Ca can increase rice antioxidant enzyme activity by modulating rice antioxidant enzyme-related genes, reducing the oxidative damage caused by salt stress.

## Conclusions

**Fig 8:**
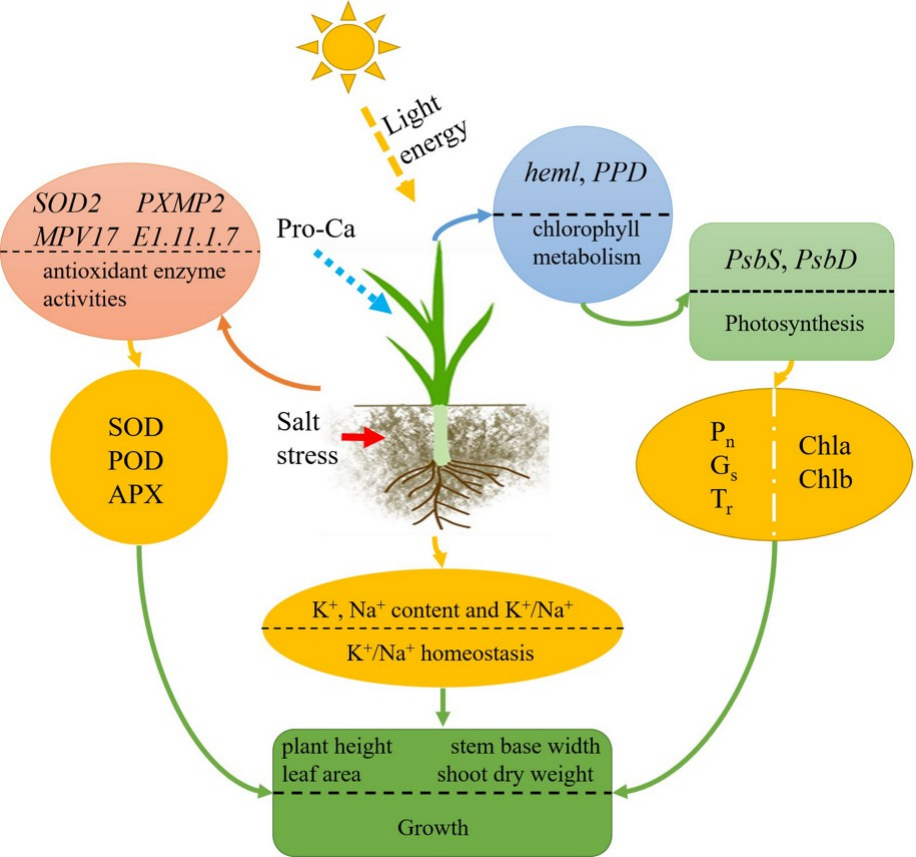
Schematic diagram of the mechanism by which prohexadione-calcium promotes rice seedlings development under salt stress by regulating antioxidant processes and photosynthesis.

Pro-Ca reduced salt stress and enhanced rice seedling growth in various ways. According to our findings, Pro-Ca reduced ROS damage by modulating the expression of antioxidant enzyme-related genes (such as *SOD2*, *PXMP2*, *MPV17*, *E1*.*11*.*1*.*7*) and enhancing antioxidant enzyme activity. Furthermore, Pro-Ca regulated the expression of photosynthesis genes (such as *PsbS*, *PsbD*) and chlorophyll metabolism genes (*heml*, *PPD*), increasing chlorophyll content and photosynthesis. At the same time, Pro-Ca regulated the content of Na^+^ and alleviated the damage of salt stress to ion balance. In conclusion, Pro-Ca promotes rice seedlings’ development under salt stress by regulating antioxidant processes and photosynthesis (Fig 8). This discovery clarified the positive role of Pro-Ca in rice salt tolerance, and improved the potential mechanism of Pro-Ca in improving rice salt tolerance.

## Supporting information

S1 Fig Effect of exogenous prohexadione-calcium (Pro-Ca) on MDA content in rice under salt stress. Abbreviations: CK, control (normal water); S, salt stress; and S + Pro-Ca, salt stress plus foliar Pro-Ca application. Values represent mean ± SE (n = 3), and different lowercase letters indicate significant differences according to Duncan’s test.
(TIF)

S1 Table Pro-Ca or salt induced expression patterns of photosynthesis, chlorophyll metabolism and antioxidant processes-related gene metabolism genes. The expression level changes of genes in each group were described as log2 fold change of FPKM. Q-Value<0.01 and |FC|>1.5.
(DOCX)

S2 Table Prime information. (DOCX)
