# Supplementary figures and images for: Physiological and transcriptome analysis reveals that prohexadione-calcium promotes rice seedling’s development under salt stress by regulating antioxidant processes and photosynthesis
Source: PLoS One. 2023 Jun 14;18(6):e0286505. doi: 10.1371/journal.pone.0286505 (PMC10266641; doi:10.1371/journal.pone.0286505)

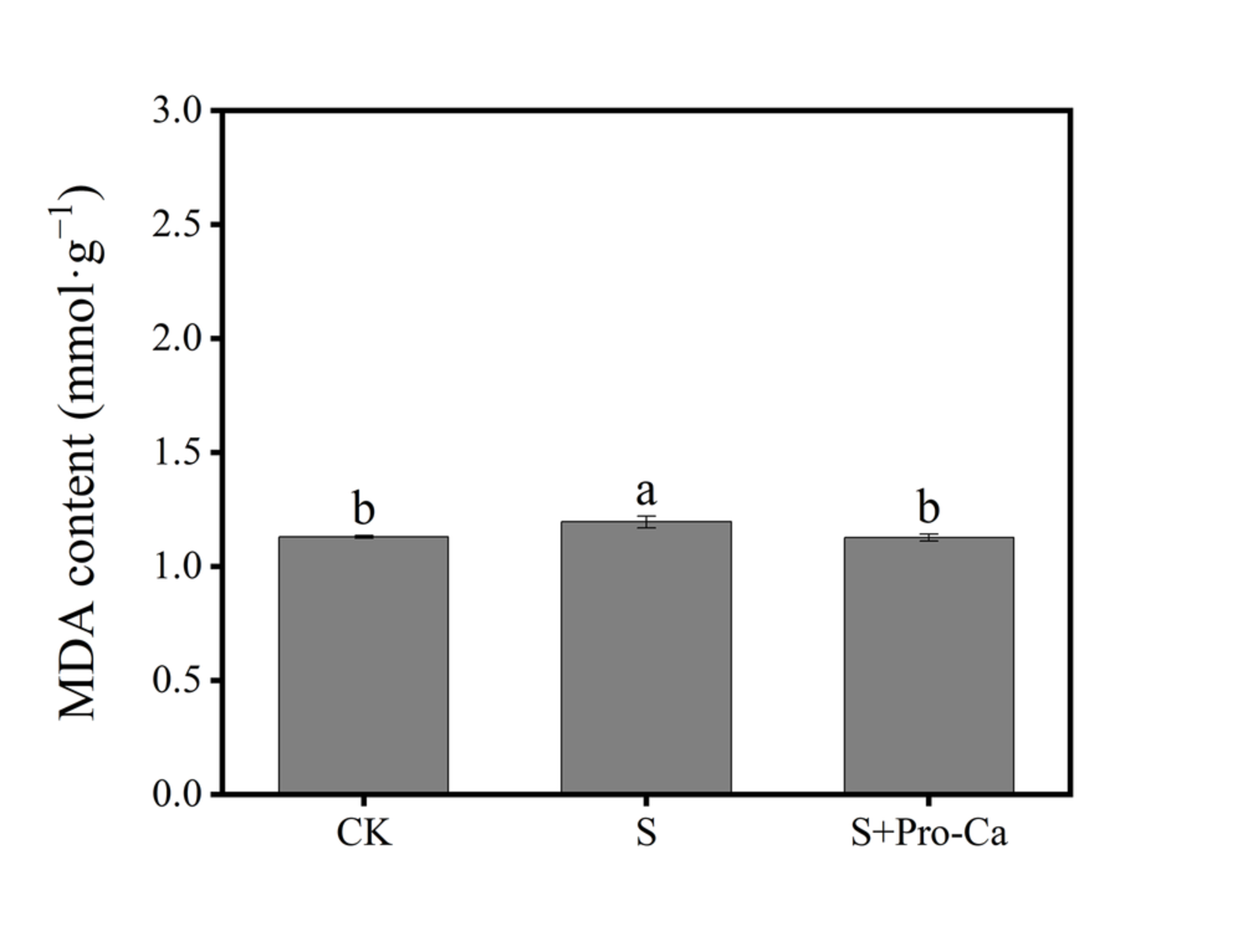

Supplement: S1 Fig — Abbreviations: CK, control (normal water); S, salt stress; and S + Pro-Ca, salt stress plus foliar Pro-Ca application. Values represent mean ± SE (n = 3), and different lowercase letters indicate significant differences according to Duncan’s test. (TIF) [file pone.0286505.s001.tif]
