# Supplementary material for: Physiological and transcriptome analysis reveals that prohexadione-calcium promotes rice seedling’s development under salt stress by regulating antioxidant processes and photosynthesis
Source: PLoS One. 2023 Jun 14;18(6):e0286505. doi: 10.1371/journal.pone.0286505 (PMC10266641; doi:10.1371/journal.pone.0286505)
Supplement: S1 Table — The expression level changes of genes in each group were described as log2 fold change of FPKM. Q-Value<0.01 and |FC|>1.5. (DOCX) [file pone.0286505.s002.docx]

**S1 Table. Pro-Ca induced expression patterns of photosynthesis, chlorophyll metabolism and antioxidant processes-related gene metabolism genes**

| **gene ID** | **Symbol** | **GO annotation** | **KEGG annotation** | **KEGG pathway annotation** |
| --- | --- | --- | --- | --- |
| ZS04g0813000 | *psb S* | chloroplast (GO:0009507) | K03542  photosystem II 22kDa protein | Photosynthesis (ko00195) |
| ZS11g0441200 | *psb D* | photosystem II (GO:0009523)  chloroplast thylakoid membrane (GO:0009535)  photosynthetic electron transport in photosystem II (GO:0009772)  chlorophyll binding (GO:0016168) | K02706  photosystem II P680 reaction center D2 protein | Photosynthesis (ko00195) |
| ZS02g0044100 | *heml* | -- | K01845  glutamate-1-semialdehyde 2,1-aminomutase | Porphyrin and chlorophyll metabolism (ko00860) |
| ZS07g0506700 | *PPD* | -- | K13544  pheophorbidase | Porphyrin and chlorophyll metabolism (ko00860) |
| ZS06g0022800 | *SOD2* | superoxide dismutase activity (GO:0004784) | K04564  superoxide dismutase, Fe-Mn family | Peroxisome (ko04146) |
| ZS11g0035400 | *PXMP2* | integral component of membrane (GO:0016021) | K13347  peroxisomal membrane protein 2 | Peroxisome (ko04146) |
| ZS12g0380700 | *MPV17* | cytoplasm (GO:0005737)  integral component of membrane (GO:0016021) | K13348  protein Mpv17 | Peroxisome (ko04146) |
| ZS06g0617800 | *E1.11.1.7* | peroxidase activity (GO:0004601)  response to oxidative stress (GO:0006979)  hydrogen peroxide catabolic process (GO:0042744) | K00430  peroxidase | Phenylpropanoid biosynthesis (ko00940) |

The expression level changes of genes in each group were described as log2 fold change of FPKM. P<0.01 FC>1.5.
