# Supplementary material for: Physiological and transcriptome analysis reveals that prohexadione-calcium promotes rice seedling’s development under salt stress by regulating antioxidant processes and photosynthesis
Source: PLoS One. 2023 Jun 14;18(6):e0286505. doi: 10.1371/journal.pone.0286505 (PMC10266641; doi:10.1371/journal.pone.0286505)
Supplement: S2 Table — (DOCX) [file pone.0286505.s003.docx]

**S2 Table. Prime information**

| **Primers** | **5'to3'** | **TM** |
| --- | --- | --- |
| *UBQ10*-F | GGTCAGTAATCAGCCAGTT | 60℃ |
| *UBQ10*-R | CCAGACACAAGTAGACATCA |  |
| *heml*-F | GCACGATTCAACAGCAAGA | 60℃ |
| *heml*-R | TGAGCCACAAGTTCATACAAC |  |
| *PPD*-F | TGGTCCTAACAGAAGAGAAGT | 60℃ |
| *PPD*-R | CACATACCTTGCTGAAGATGA |  |
| *SOD2*-F | TTCTATTGGCGATCTATGC | 60℃ |
| *SOD2*-R | CCAAAGTCCCTGTTGATAA |  |
| *MPV17*-F | GTTCATTTACAGCCGCTAGT | 63℃ |
| *MPV17*-R | AACTCGTGGTCACATCAAC |  |
| *E1.11.1.7*-F | GACCATGAACTTCACCGA | 60℃ |
| *E1.11.1.7*-R | CCCTGACAACCAGATGAG |  |
